# Accurately Deciphering Tissue Heterogeneity From Spatial Multi‐Modal and Multi‐Omics With STransformer

**DOI:** 10.1002/advs.75969

**Published:** 2026-06-09

**Authors:** Xingyi Li, Jialuo Xu, Gaoyuan Du, Xiangting Jia, Dongmin Zhao, Chunyan Zhou, Kexin Xiao, Jia Gu, Junnan Zhu, Xuequn Shang

**Affiliations:** ^1^ School of Computer Science Northwestern Polytechnical University Xi'an Shaanxi China; ^2^ Shenzhen Research Institute of Northwestern Polytechnical University Shenzhen Guangdong China; ^3^ Faculty of Data Science City University of Macau Macau China; ^4^ State Key Laboratory of Multimodal Artificial Intelligence Systems, Institute of Automation Chinese Academy of Sciences Beijing China

**Keywords:** graph neural networks, spatial multi‐modal and multi‐omics, tissue heterogeneity, transformer

## Abstract

Advances in spatially resolved technologies enable the simultaneous acquisition of diverse data modalities within a tissue slice while preserving critical spatial context, which presents unprecedented opportunities to decipher intricate tissue heterogeneity. However, existing computational approaches lack the intrinsic flexibility to universally process both spatial multi‐modal and multi‐omics data. Here, we introduce STransformer, a unified deep learning framework designed to seamlessly accommodate a comprehensive landscape of spatial data. By simultaneously capturing short‐range cellular interactions and tissue‐wide semantic patterns, it extracts robust representations to accurately dissect complex tissue heterogeneity. Systematic evaluations across diverse species, tissue types, and data modalities highlight its profound versatility. For spatial multi‐modal data, STransformer delineates intricate anatomical structures in the human cortex, uncovers pathological mechanisms in Alzheimer's disease, and characterizes dynamic spatiotemporal developmental trajectories during chicken cardiogenesis. Scaling to spatial multi‐omics data, STransformer synergizes spatial transcriptomic and proteomic profiles to decipher intricate immune microenvironments within the human tonsil, and jointly analyzes spatial epigenomic and transcriptomic data to infer regulatory mechanisms in the mouse embryonic brain. Consequently, STransformer serves as a highly versatile and robust analytical framework for advancing our understanding of tissue heterogeneity and disease pathogenesis.

## Graphical abstract

STransformer is a unified deep learning framework designed to seamlessly accommodate a comprehensive landscape of spatial data. By simultaneously capturing short‐range cellular interactions and tissue‐wide semantic patterns, it extracts robust representations to accurately dissect complex tissue heterogeneity. Systematic evaluations across diverse species, tissue types, and data modalities highlight its profound versatility.

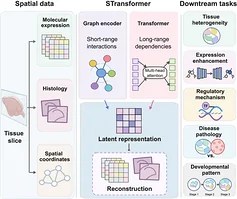

## 1 Introduction

Tissue heterogeneity arises from its diverse cellular composition and the precisely organized spatial relationships among cells. Unraveling the intricacies of tissue heterogeneity necessitates the accurate identification of functional spatial regions, which are essential for resolving complex tissue organization and disease mechanisms [1, 2, 3].

The rapid advancement of high‐throughput spatial technologies has provided unprecedented opportunities to characterize complex tissues by enabling the acquisition of diverse data types while preserving spatial context. Specifically, platforms such as 10× Visium [4] facilitate the generation of spatial multi‐modal data by simultaneously capturing histological images, gene expression profiles, and spatial coordinates. Furthermore, the evolution of spatial multi‐omics technologies allows for the concurrent profiling of multiple molecular layers within an identical tissue slice. For instance, 10x Visium RNA with protein co‐profiling technology can now simultaneously capture the spatial transcriptome and protein abundance, while sequencing‐based technologies like MISAR‐seq [5] enable the joint analysis of the spatial epigenome and transcriptome.

The increasing accumulation of spatial multi‐modal and multi‐omics data has driven the development of computational methods for dissecting tissue heterogeneity (a summary of representative methods is provided in Table S1). Nevertheless, most existing studies primarily focus on unimodal data, such as STAGATE [6], SEDR [7], GraphST [8], and stDCL [3]. By constructing spatial graphs exclusively from gene expression and spatial coordinates, these methods neglect the synergistic information from other modalities or omics, thereby limiting the characterization of complex tissue heterogeneity. Although image‐guided methods like stLearn [9], DeepST [10], and STAIG [11] attempt to incorporate morphology, they predominantly treat image‐derived features as auxiliary information rather than engaging in deep multi‐modal fusion. Concurrently, spatial multi‐omics modeling frameworks like SpatialGlue [12], COSMOS [13], SpaMV [14], and soFusion [15] have demonstrated efficacy by leveraging Graph Neural Networks (GNNs). However, while GNNs are highly effective at aggregating short‐range cellular interactions within local neighborhoods, they are inherently limited in capturing long‐range dependencies. Consequently, there is significant potential to enhance the performance of deciphering complex tissue architectures by integrating global context.

In this study, we propose STransformer, a versatile deep learning framework designed to seamlessly integrate diverse spatial data, ranging from spatial multi‐modal (e.g., gene expression, spatial coordinates, and histological images) to multi‐omics (e.g., spatial epigenome‐transcriptome and transcriptome‐proteome). By synergistically integrating Graph Convolutional Networks (GCNs) with a Transformer architecture, STransformer simultaneously captures information about local cellular niches and global tissue organization. Specifically, GCNs are employed to encode intrinsic features of heterogeneous modalities based on spatial neighborhoods, while the Transformer utilizes self‐ and cross‐attention to enable deep cross‐modal integration across the tissue slice. By modeling both short‐range cellular interactions and tissue‐wide structural dependencies, STransformer generates spatially aware and globally coherent latent representations, enabling the accurate characterization of tissue heterogeneity.

We conduct comprehensive evaluations of STransformer across a diverse range of species, tissue types, and molecular profiles. When applied to spatial multi‐modal data, STransformer accurately delineates complex anatomical structures in the human dorsolateral prefrontal cortex, and dissects cellular communication networks in Alzheimer's disease, elucidating its underlying pathological mechanisms. Extending to spatial multi‐omics data, STransformer synergizes spatial transcriptomic and proteomic profiles to decipher intricate immune microenvironments within the human tonsil, and jointly analyzes spatial epigenomic and transcriptomic data to infer potential regulatory mechanisms in the mouse embryonic brain. Finally, utilizing time‐series spatial data of the chicken embryonic heart, STransformer successfully captures the dynamic spatiotemporal trajectories of cardiogenesis.

## 2 Results

### 2.1 Overview of STransformer

**FIGURE 1:**
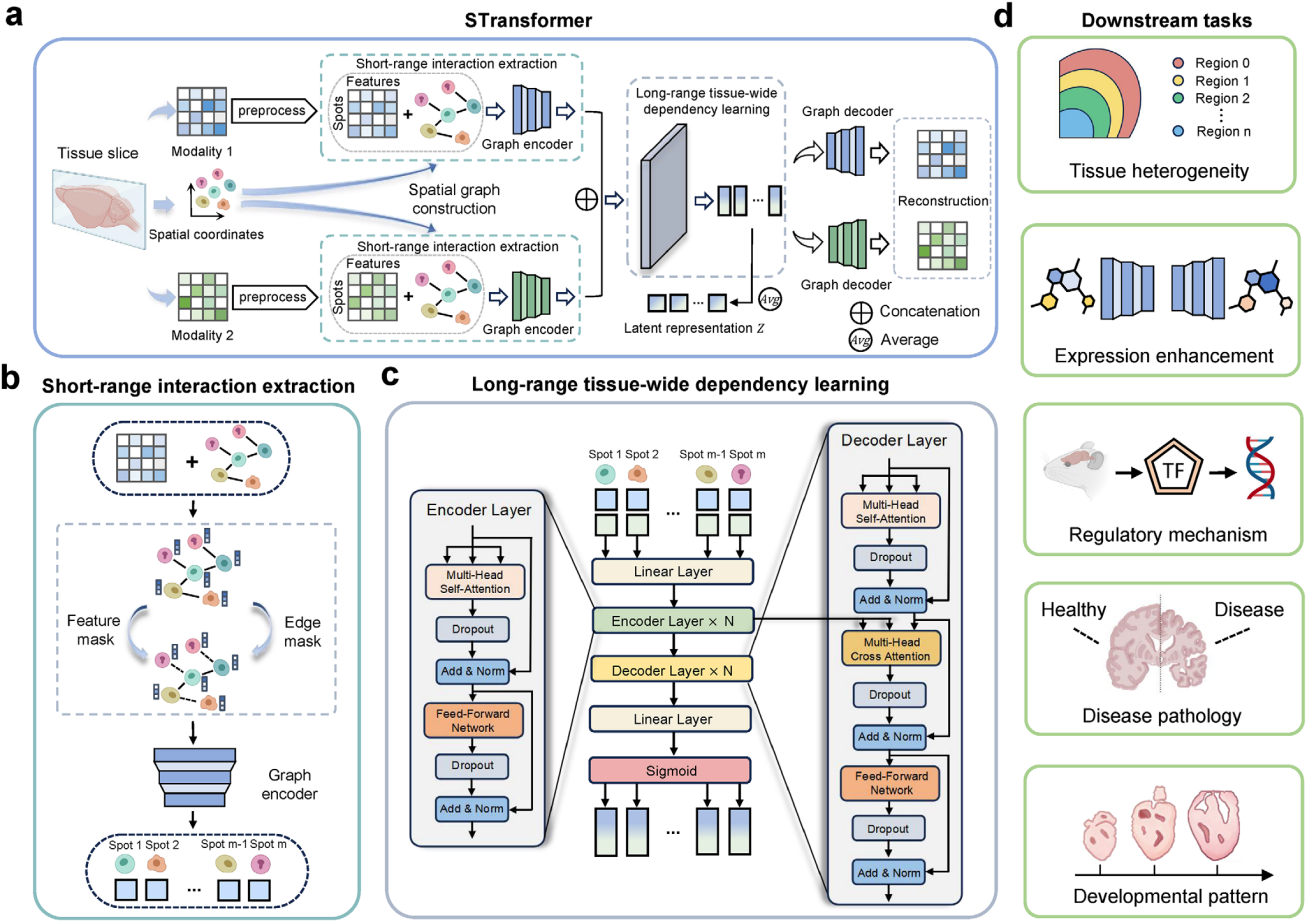
Overview of STransformer. (a) Schematic workflow of STransformer. STransformer is a versatile deep learning framework that accepts both spatial multi‐modal and multi‐omics data as input. After data preprocessing, spatial graphs are constructed based on spatial coordinates, while molecular expression profiles are used as node features. The framework then utilizes GCN‐based graph encoders to extract short‐range cellular interactions, followed by a Transformer that captures long‐range tissue‐wide dependencies, yielding comprehensive latent spatial representations. Finally, the architecture incorporates graph decoders to perform molecular expression reconstruction, encouraging the preservation of essential biological information. (b) Short‐range interaction extraction. Given the spatial graph, a masked graph autoencoder strategy is adopted in which random masking is applied to node features and edges. GCN‐based graph encoders then aggregate neighborhood information from the masked graphs to learn modality‐specific representations that capture localized cellular interactions. (c) Long‐range tissue‐wide dependency learning. Concatenated modality‐specific representations are fed into a Transformer architecture. Through self‐attention and cross‐attention mechanisms, it captures long‐range dependencies and macroscopic spatial organization to construct latent spatial representations. (d) Downstream tasks. The latent spatial representations support various analytical tasks, including tissue heterogeneity dissection, molecular expression enhancement, regulatory mechanism inference, pathological state analysis, and developmental pattern discovery.

STransformer is a versatile deep learning framework that synergistically integrates GCNs with a Transformer architecture for the accurate dissection of tissue heterogeneity (Figure 1a). It is capable of processing a comprehensive landscape of spatial inputs, including spatial multi‐modal and multi‐omics data. We collectively refer to these two data types as modalities hereafter for brevity. After data preprocessing, spatial graphs are constructed based on spatial coordinates, while molecular expression profiles are used as node features. STransformer employs GCN‐based graph encoders to extract short‐range spatial interactions within spatial graphs (Figure 1b). These encoders aggregate neighborhood information from spatial graphs, yielding modality‐specific representations that capture localized cellular interactions. The resulting modality‐specific representations are then concatenated and processed by a Transformer architecture to learn long‐range tissue‐wide dependencies (Figure 1c). Through self‐attention and cross‐attention mechanisms, the Transformer captures cross‐modal relationships and macroscopic spatial organization across the tissue, generating latent representations that reflect both short‐range cellular interactions and long‐range spatial dependencies. To further preserve biologically meaningful information during representation learning, STransformer employs graph decoders to reconstruct molecular expression profiles from the learned representations, which serve as a reconstruction objective that regularizes the representation learning process. The learned representations subsequently enable multiple downstream analyses (Figure 1d), encompassing spatial architecture delineation, molecular expression enhancement, regulatory mechanism inference, disease pathology characterization, and the discovery of spatiotemporal developmental patterns.

### 2.2 Dissecting Tissue Heterogeneity in Human Dorsolateral Prefrontal Cortex From Spatial Multi‐Modal Data

To comprehensively assess the capability of STransformer in identifying spatial domains, we employ the human dorsolateral prefrontal cortex (DLPFC) dataset from the 10× Visium platform, which contains spatial transcriptomic profiles and corresponding histological images. This dataset comprises 12 slices from three donors and includes six cortical layers (Layer 1 to Layer 6) and white matter (WM) [16]. We benchmark STransformer against seven state‐of‐the‐art spatial domain identification methods (SEDR [7], STAGATE [6], GraphST [8], stLearn [9], STAIG [11], DeepST [10], and stDCL [3]), and assess clustering performance using the Adjusted Rand Index (ARI) [17], as detailed in Note S1.

**FIGURE 2:**
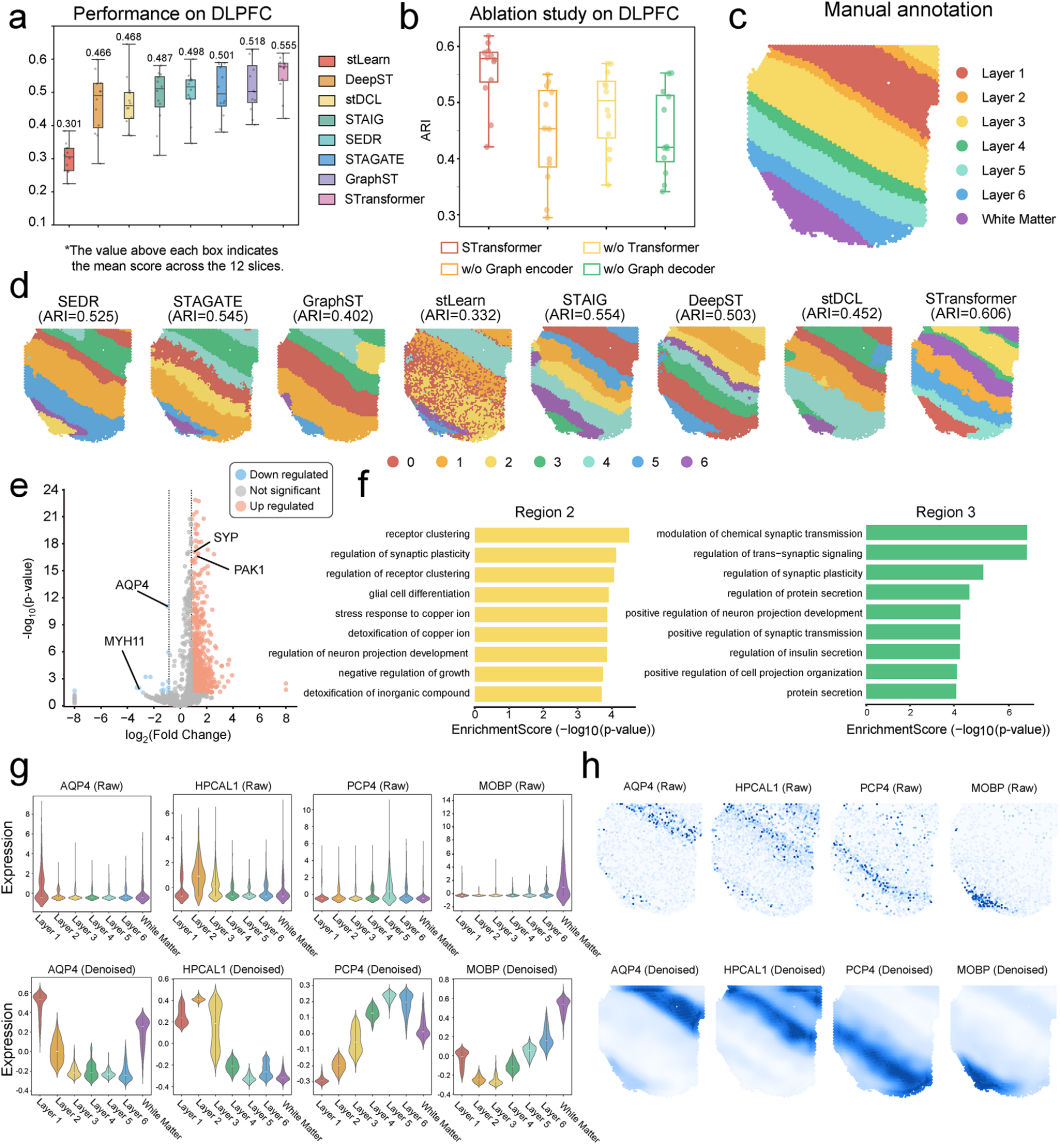
Dissecting tissue heterogeneity in human dorsolateral prefrontal cortex from spatial multi‐modal data. (a) Quantitative evaluation of spatial clustering performance across all 12 DLPFC slices. Boxplots illustrate the ARI scores for STransformer and seven competing methods. Box limits indicate the interquartile range (IQR), and the value above each box denotes the mean score. (b) Ablation analysis evaluating the contribution of individual components in STransformer across all slices. Boxplots illustrate the ARI scores of the full model and its ablated variants. (c) Manual annotations of the cortical layers and white matter (WM) in slice 151507. (d) Cortical layers identified by STransformer and competing methods in slice 151507. (e) Volcano plot displaying differentially expressed genes (DEGs) between domain 2 (corresponding to Layer 1) and domain 3 (corresponding to Layer 2) in slice 151507. Red and blue points represent genes significantly upregulated or downregulated in Layer 2 relative to Layer 1, respectively. (f) GO enrichment analysis of domain 2 (corresponding to Layer 1) and domain 3 (corresponding to Layer 2) identified by STransformer in slice 151507, highlighting the most significantly enriched terms. (g) Violin plots displaying raw and reconstructed expression distributions of layer‐specific marker genes in slice 151507 (*AQP4*: Layer 1; *HPCAL1*: Layer 2; *PCP4*: Layer 5; *MOBP*: White Matter). (h) Raw and STransformer‐denoised expression patterns of layer‐specific marker genes in slice 151507.

Overall, STransformer outperforms competing methods across all 12 slices, achieving the highest scores in both median (Figure 2a and Figures S1–S3, ARI = 0.578) and mean metrics (ARI = 0.555). This highlights its superior robustness and reliable performance in spatial domain identification. To systematically evaluate the contribution of each architectural component within STransformer, we perform comprehensive ablation studies (Figure 2b and Table S2). The complete model consistently outperforms all ablated variants across all tissue slices. Specifically, eliminating either the graph encoder or the graph decoder results in a substantial performance degradation, confirming their indispensable roles in capturing short‐range cellular interactions and generating robust latent representations. Similarly, the omission of the Transformer module noticeably diminishes accuracy, indicating that long‐range spatial dependencies provide the critical contextual information essential for deciphering tissue heterogeneity. These results demonstrate that precise spatial region identification relies heavily on the joint modeling of localized cellular interactions and tissue‐wide global organization.

In addition, STransformer achieves the highest ARI of 0.606 in slice 151507, outperforming all competing methods (Figure 2c,d). Importantly, STransformer accurately distinguishes the complex sandwich‐like structure (Layer 1–Layer 2–Layer 1), whereas GNN‐only methods such as STAGATE and GraphST fail to fully resolve this structure, highlighting the importance of jointly capturing both short‐range cellular interactions and long‐range tissue‐wide dependencies. We then perform differential expression (DE) analysis (detailed in Note S3) between domain 2 (corresponding to Layer 1) and domain 3 (corresponding to Layer 2). The volcano plot (Figure 2e) reveals the upregulation of synaptic regulators (*SYP* and *PAK1*) in Layer 2 [18, 19], aligning with the functional role of the layer in intracortical information processing [16, 20]. Conversely, genes such as *AQP4* and *MYH11* are enriched in Layer 1, reflecting the dense astrocytic end‐feet of the glia limitans and vascular elements characteristic of the cortical molecular layer [16, 21]. Furthermore, Gene Ontology (GO) enrichment analysis (detailed in Note S4) delineates the functional roles of these two layers (Figure 2f). Layer 1 is predominantly enriched in receptor clustering, synaptic plasticity regulation and glial cell differentiation, reflecting its function as a synaptic input compartment characterized by a glia‐rich microenvironment [22]. Layer 2 is enriched in the modulation of chemical synaptic transmission and protein secretion, suggesting its potential involvement in superficial‐layer intracortical communication [23]. Collectively, these findings validate the capacity of STransformer to dissect tissue architecture into biologically coherent domains.

Furthermore, the inherent technical noise in spatial expression data often obscures critical biological patterns, posing a significant challenge to the accurate characterization of tissue heterogeneity. To mitigate this, STransformer enhances spatial gene expression profiles by decoding informative latent representations. In slice 151507, we evaluate the expression patterns of canonical layer markers before and after denoising. The results reveal that the enhanced gene expression exhibits pronounced layer‐specific enrichment and reveals biologically meaningful patterns (Figure 2g,h). For instance, the denoised patterns of the Layer 1‐specific marker *AQP4* and the Layer 2‐specific marker *HPCAL1* exhibit reinforced spatial specificity and are consistent with previously reported smFISH patterns in the human DLPFC. In addition, the other genes, *PCP4* and *MOBP*, are also consistent with their reported roles as Layer 5 and white matter markers, respectively [16].

### 2.3 Revealing Pathological Mechanisms in Alzheimer's Disease From Spatial Multi‐Modal Data

**FIGURE 3:**
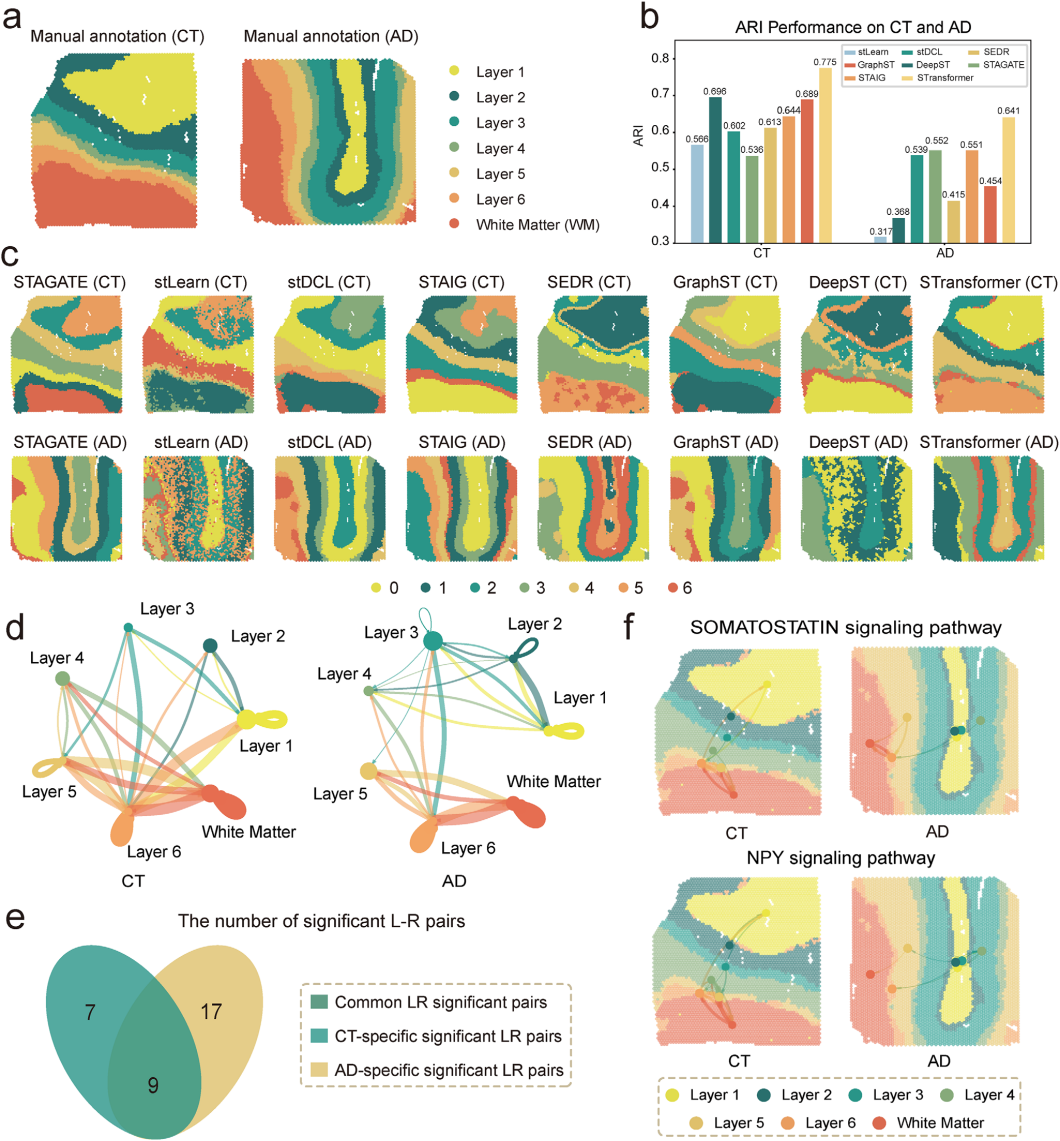
Revealing pathological mechanisms in Alzheimer's disease from spatial multi‐modal data. (a) Manual annotations of cortical layers and white matter in the MTG dataset for healthy control (CT) and Alzheimer's disease (AD) slices. (b) Quantitative evaluation of clustering performance on CT and AD slices, with bar plots illustrating the ARI scores for STransformer and competing methods. (c) Spatial regions identified by STransformer and competing methods in CT and AD slices. (d) Circle plots illustrating the interlaminar communication patterns in CT and AD groups. (e) Venn diagram displaying the overlap and specificity of significant L‐R pairs in CT and AD groups. (f) Spatial visualization of key homeostatic signaling pathways (SOMATOSTATIN and NPY) in CT and AD groups.

To delineate the pathological mechanisms underlying Alzheimer's disease, we apply STransformer to the 10× Visium human middle temporal gyrus (MTG) dataset [24], which contains spatial transcriptomic profiles and corresponding histological images. This dataset encompasses tissue slices from both healthy controls (CT) and Alzheimer's disease (AD) patients, featuring manual annotations of six cortical layers (Layer 1–Layer 6) and white matter (Figure 3a). Quantitatively, STransformer demonstrates superior identification performance on both the CT (ARI = 0.775) and AD (ARI = 0.641) slices (Figure 3b). In particular, STransformer accurately dissects the laminar cortical architecture, showing high concordance with the manual annotations (Figure 3c).

We also systematically characterize the pathological shifts in cellular communication patterns induced by AD (Note S5). Our analysis reveals a profound perturbation of the homeostatic interlaminar signaling within the MTG (Figure 3d). Specifically, compared to the CT group, the AD group shows suppressed interlaminar connectivity originating from Layer 6. Conversely, communication among Layer 1 through Layer 4 is substantially amplified. Moreover, the AD group exhibits enriched self‐loop communication patterns, implying a heightened state of autocrine activity across the cortical layers.

Furthermore, we analyze the alterations in significant ligand‐receptor (L‐R) pairs between the CT and AD groups (Figure 3e and Tables S4 and S5). In the CT group, we detect 16 significant L‐R pairs, which are predominantly associated with the regulation of excitation/inhibition balance (e.g., SST‐SSTR1 in the SOMATOSTATIN pathway [25]) and neuroprotection (e.g., NPY‐NPY1R in the NPY pathway [26, 27]). In contrast, in the AD group, we detect a greater number of significant L‐R pairs (26 pairs). The emergence of inflammatory chemokine interactions (e.g., CXCL10‐ACKR1 in the CXCL pathway [28] and CCL2‐ACKR1 in the CCL pathway [29]) indicates a transition from homeostatic signaling to a neuroinflammatory microenvironment. Finally, we visualize key signaling pathways that are essential for maintaining homeostasis (e.g., SOMATOSTATIN [25] and NPY [27] in Figure 3f). Notably, these pathways are markedly altered in the AD group relative to the CT group, suggesting a disruption of homeostatic signaling architecture in the diseased state.

### 2.4 Deciphering Intricate Tissue Architecture in Human Tonsil From Spatial Transcriptome‐Proteome Data

**FIGURE 4:**
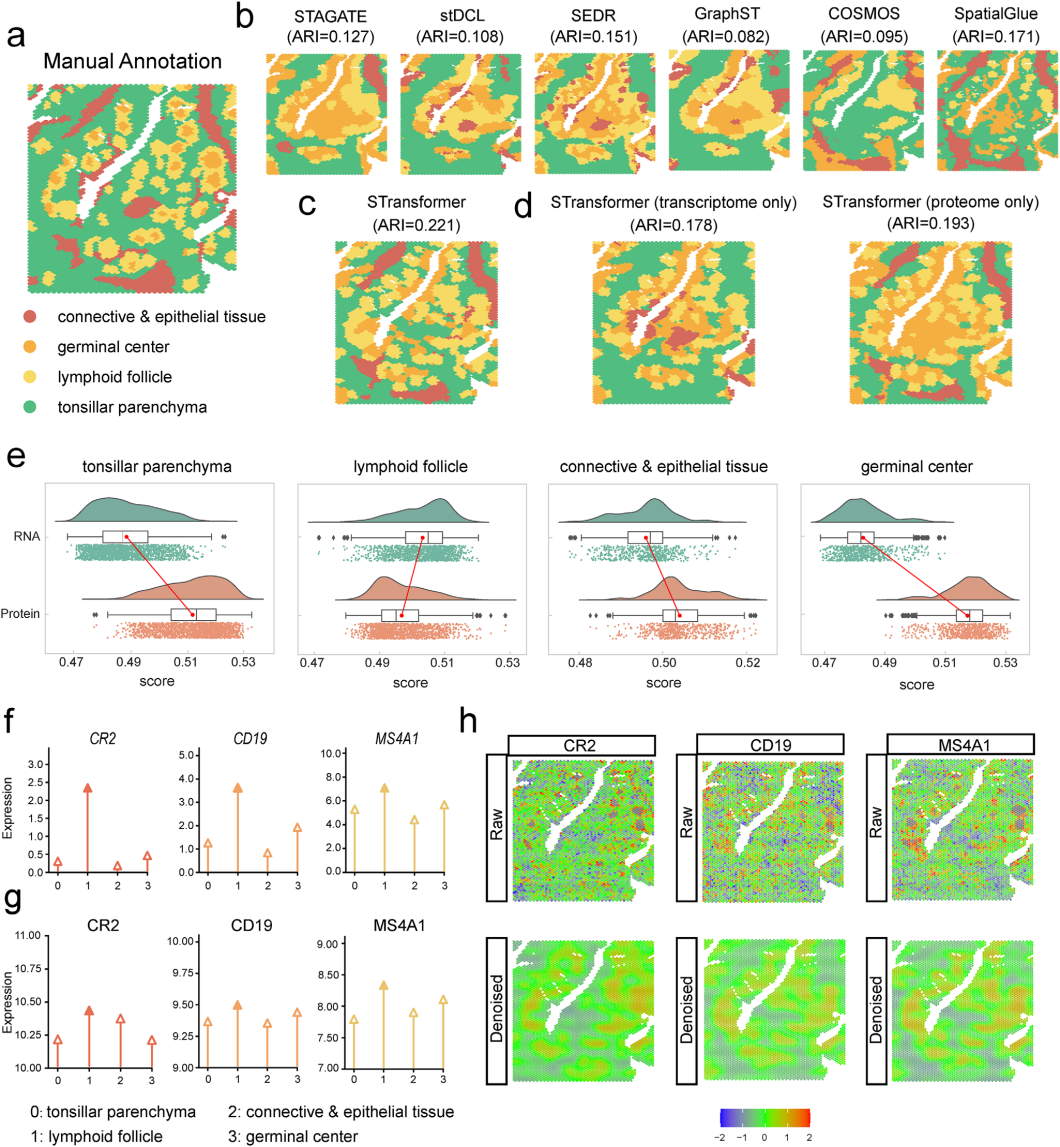
Deciphering intricate tissue architecture in human tonsil from spatial transcriptome‐proteome data. (a) Manual annotations of the four tissue regions in the human tonsil dataset. (b) Spatial regions identified by competing methods in the human tonsil, accompanied by their corresponding ARI scores. (c) Spatial regions identified by STransformer in the human tonsil, accompanied by the corresponding ARI score. (d) Ablation study comparing the clustering performance (ARI) of STransformer using unimodal inputs (transcriptome‐only or proteome‐only). (e) Raincloud plots illustrating the adaptive contribution of transcriptomics and proteomics across different tissue regions. Red dots denote the mean modality contribution scores, and red lines connect the mean values between the two modalities. (f) Lollipop plots displaying expression enrichment of canonical B‐cell marker genes in the identified lymphoid follicle regions. The solid arrows indicate the highest expression value for each marker. (g) Lollipop plots displaying expression intensity of surface protein markers in the identified lymphoid follicle regions. The solid arrows indicate the highest expression value for each marker. (h) Raw and STransformer denoised protein expression levels.

We next extend our validation to a spatial multi‐omics dataset of the human tonsil, which is generated using the 10× Visium with protein co‐profiling technology. This dataset comprises spatial transcriptomic and proteomic profiles with four manually annotated tissue regions, including connective & epithelial tissue, germinal center, lymphoid follicle, and tonsillar parenchyma (Figure 4a).

First, we evaluate the capacity of STransformer to delineate intricate tissue architectures by integrating spatial multi‐omics information. In this assessment, STransformer yields superior clustering accuracy (ARI = 0.221), significantly outperforming all competing methods. To further quantify the benefit of multi‐omics integration, we evaluate STransformer with single‐omics inputs (Figure 4d). The results show that STransformer with integrated multi‐omics data consistently outperforms its transcriptome‐only (ARI = 0.178) and proteome‐only (ARI = 0.193) ablations. Notably, STransformer utilizing single‐omics data still surpasses the performance of competing methods utilizing multi‐omics data. This result indicates that by jointly capturing local cellular interactions and long‐range tissue dependencies, STransformer derives exceptionally robust latent representations, enabling highly accurate structural dissection even when relying on single‐omics data. We also assess the region‐specific contributions of each omics modality by examining the modality weights (Figure 4e). In most anatomical regions, the transcriptomic modality exhibits a dominant contribution. Conversely, for the lymphoid follicle region, the proteomic modality demonstrates superior discriminative power and is consequently assigned a higher weight.

Given that the human tonsil is a primary lymphoid organ, the lymphoid follicle represents a biologically significant region for intense B‐cell maturation and differentiation [30, 31]. Therefore, we examine the transcriptomic and proteomic expression of B‐cell signatures in this region. We observe that canonical B‐cell marker genes [30] (*CR2*, *CD19*, *MS4A1*) are highly expressed within the identified follicular regions (Figure 4f). Crucially, this transcriptional signature aligns with the proteomic data (Figure 4g), as surface protein markers (CR2, CD19, MS4A1) show maximal abundance in the follicles. These findings validate STransformer's capacity to accurately capture the intrinsic coupling between multi‐omics modalities. Furthermore, a comparison of the raw and STransformer‐denoised protein intensities of these key markers demonstrates that our model not only mitigates technical background noise but also improves the clarity of the spatial patterns (Figure 4h).

### 2.5 Resolving Mouse Embryonic Brain Structures from Spatial Epigenome‐Transcriptome Data

**FIGURE 5:**
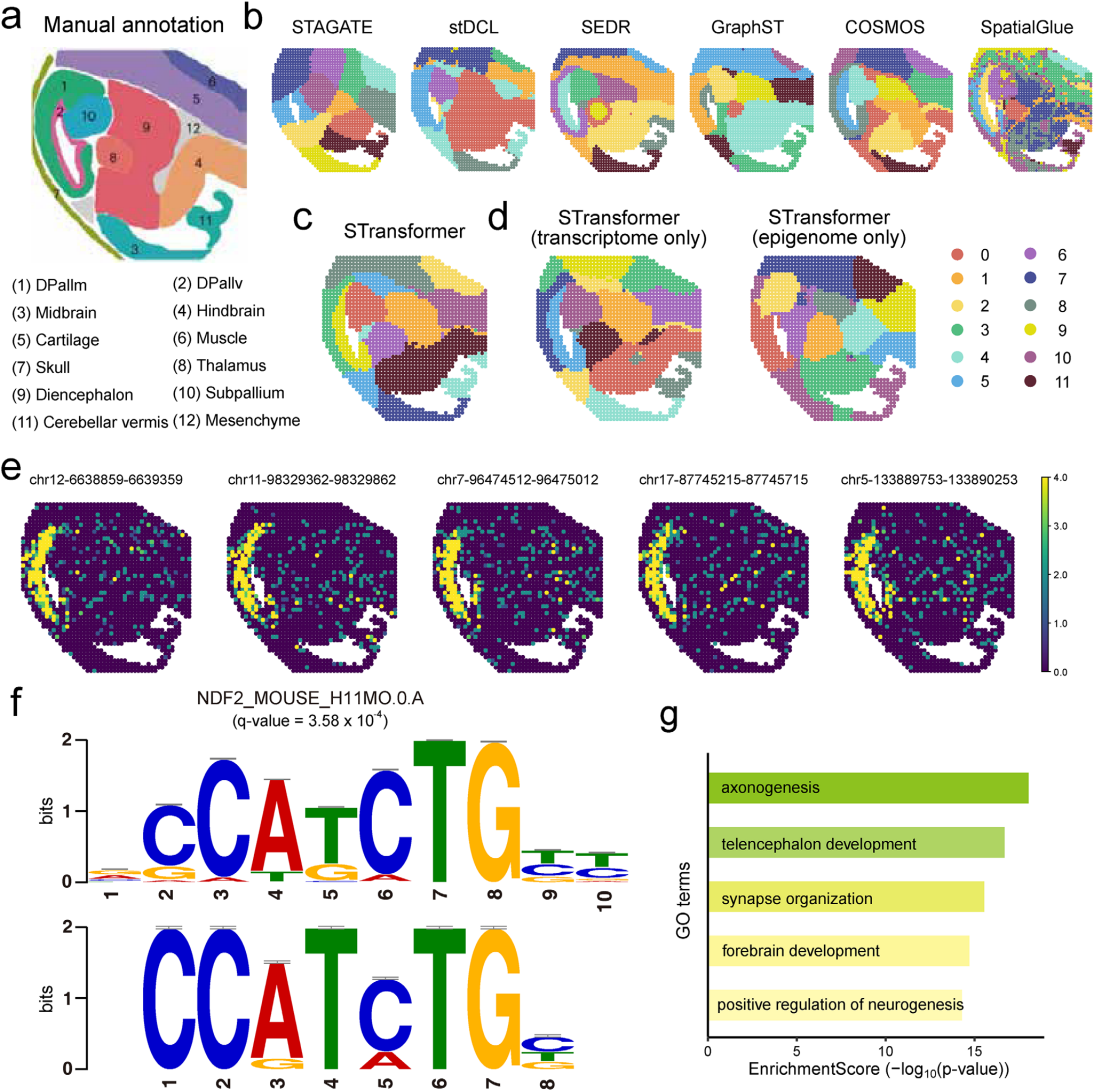
Resolving embryonic mouse brain structures from spatial epigenome‐transcriptome data. (a) Manual annotations of the twelve tissue regions in the mouse embryonic brain (E15.5) dataset. (b) Visualization of spatial regions identified by competing methods in the mouse embryonic brain. (c) Visualization of spatial regions identified by STransformer in the mouse embryonic brain. (d) Ablation study comparing the clustering performance of STransformer using unimodal inputs (transcriptome‐only or epigenome‐only). (e) Top five peaks related to the dorsal pallium. Colors represent peak accessibility. (f) Motif enrichment analysis for the dorsal pallium region, showing the sequence alignment of the known NDF2 reference (top) with the *de novo* discovered motif (bottom). (g) GO enrichment analysis for the dorsal pallium region identified by STransformer, showing the top five significantly enriched terms.

To demonstrate the versatility of STransformer across diverse spatial multi‐omics modalities, we extend our evaluation to a mouse brain epigenome‐transcriptome dataset. The E15.5 mouse embryonic brain slice [5] comprises 12 manually annotated tissue regions (Figure 5a). This dataset is acquired using MISAR‐seq [5], an advanced sequencing technology that simultaneously profiles gene expression and chromatin accessibility (ATAC) within the same tissue architecture.

We first assess the capacity of STransformer to delineate tissue heterogeneity in the mouse embryonic brain. Quantitative analysis reveals that STransformer achieves concordance with manual annotations and outperforms other methods in resolving anatomical regions (Figure 5b,c). Second, we compare the multi‐omics framework with unimodal (transcriptome‐ or epigenome‐only) variants (Figure 5d). This comparison confirms that integrating epigenetic and transcriptomic data yields substantial performance gains.

To elucidate the spatially resolved epigenetic regulatory mechanisms governing specific anatomical regions of the mouse embryonic brain, we specifically characterize the accessible chromatin landscape of the dorsal pallium (the DPall region, comprising DPallm and DPallv), a principal subdivision of the embryonic forebrain involved in cortical development. The top‐related chromatin accessibility peaks of the DPall region identified by STransformer exhibit distinctly localized spatial patterns (Figure 5e, Note S6). Subsequent motif enrichment analysis (Note S7) of the top 500 peaks is performed to infer the upstream transcriptional regulators associated with these accessible chromatin sites, revealing a highly significant enrichment of the “NDF2_MOUSE” motif (Figure 5f). The transcription factor NDF2 (*Neurod2*) is known to act upstream to drive neuronal differentiation and serves as a key regulator for neocortical development [32]. Consistent with the biological function of this enriched motif, GO enrichment analysis (Note S4) of the DPall‐associated genes highlights a robust enrichment of associated neurodevelopmental processes, including axonogenesis, synapse organization, and positive regulation of neurogenesis (Figure 5g).

### 2.6 Unraveling Spatiotemporal Heterogeneity in Chicken Heart Development From Multi‐Modal Data

**FIGURE 6:**
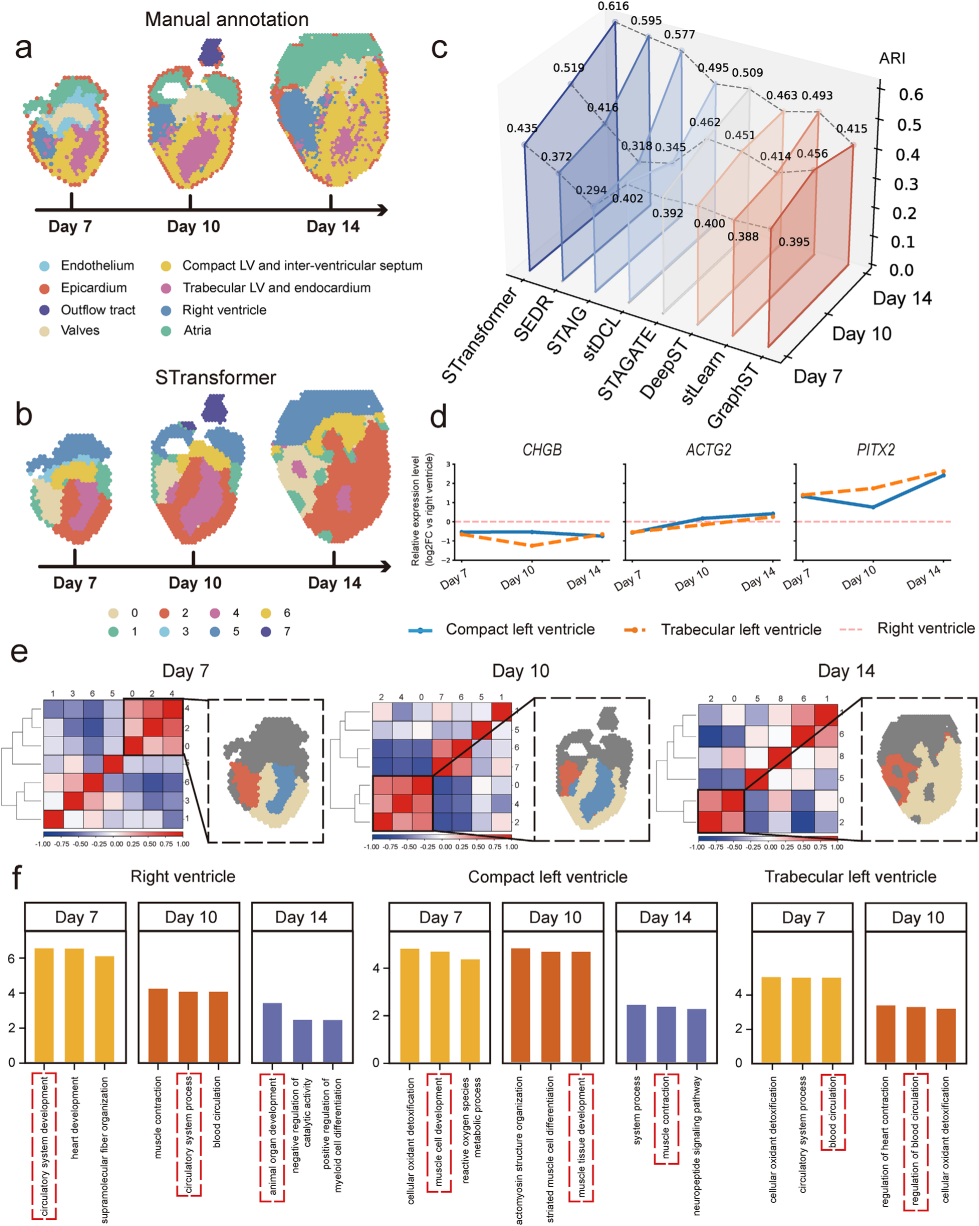
Unraveling spatiotemporal heterogeneity in chicken heart development from multi‐modal data. (a) Manual annotations of tissue regions in the chicken embryonic heart dataset across three developmental stages (Day 7, Day 10, and Day 14). (b) Visualization of spatiotemporal tissue organization identified by STransformer across three developmental stages (Day 7, Day 10, and Day 14). (c) Quantitative evaluation of clustering performance across developmental stages, with a 3D ribbon plot illustrating the ARI scores for STransformer and competing methods. (d) Temporal expression trends of key genes (*CHGB*, *ACTG2*, and *PITX2*) in the compact left ventricle and trabecular left ventricle relative to the right ventricle. (e) Correlation matrices of spatial regions identified by STransformer across three developmental stages (Day 7, Day 10, and Day 14). (f) Gene enrichment analysis of the three ventricles (right ventricle, compact left ventricle, and trabecular left ventricle) across developmental stages, showing the top three significantly enriched terms.

To unravel the complex spatiotemporal heterogeneity of tissue organization during cardiogenesis, we apply STransformer to a time‐series dataset of the chicken embryonic heart generated from the 10× Visium platform [33], which includes spatial transcriptomic profiles and corresponding histological images. This dataset spans three critical developmental stages (Day 7, Day 10, and Day 14), providing consecutive slices with regional annotations (Figure 6a). In this analysis, STransformer resolves spatial regions that exhibit high concordance with anatomical ground truth, thereby effectively capturing the dynamic evolution of tissue organization throughout development (Figure 6b). Quantitatively, STransformer consistently outperforms state‐of‐the‐art methods across all time points (Figure 6c and Figure S4), yielding high performance at Day 7 (ARI = 0.435), Day 10 (ARI = 0.519), and Day 14 (ARI = 0.616).

To assess the biological interpretability of the spatial regions identified by STransformer, we analyze the temporal expression trends of key known genes associated with ventricular maturation (Figure 6d and Note S8). The results demonstrate that *CHGB* is consistently enriched in the STransformer‐identified right ventricle throughout developmental stages, with *ACTG2* exhibiting higher expression in this region at Day 7, consistent with early ventricular developmental processes [33]. Conversely, *PITX2*, a critical gene contributing to organ laterality, maintains high expression in the identified left ventricles from Day 7 to Day 14, aligning with its established role in cardiac asymmetric morphogenesis [34, 35]. The strong concordance between these spatiotemporal expression trends and established biological knowledge firmly substantiates the efficacy of STransformer in resolving complex, biologically meaningful spatiotemporal dynamics.

Next, we analyze correlations between STransformer‐identified regions (Figure 6e, Note S9). During early development at Day 7 and Day 10, the compact left ventricle, the trabecular left ventricle, and the right ventricle exhibit high concordance. However, by Day 14, this correlation landscape undergoes a shift, retaining a strong association exclusively between the compact left ventricle and the right ventricle. This dynamic transition reflects the process of ventricular compaction during late‐stage cardiogenesis, during which the trabecular myocardium is progressively integrated into the compact layer [33]. Collectively, these observations indicate that STransformer captures not only the spatial architecture of ventricular regions but also the temporal evolution of inter‐regional relationships during cardiac maturation.

Furthermore, GO enrichment analysis (Note S4) of the STransformer‐identified regions reveals clear developmental trajectories across the chronological stages (Figure 6f). Taking the compact left ventricle as an example, we observe a distinct temporal progression: from early morphogenesis at Day 7 (e.g., “muscle cell development”), advancing to structural tissue formation at Day 10 (e.g., “muscle tissue development”), and culminating in functional execution by Day 14 (e.g., “muscle contraction”). Similar stepwise structural and functional maturation processes are consistently observed in the right ventricle and trabecular left ventricle. These findings conclusively demonstrate that the spatial regions delineated by STransformer faithfully capture the underlying temporal continuity of tissue development.

## 3 Discussion

The rapid evolution of spatial technologies has generated an unprecedented wealth of spatial multi‐modal and multi‐omics data. However, formulating a unified computational framework capable of flexibly accommodating these distinct data types remains challenging. In addition, most existing computational methods predominantly rely on GNNs for local neighborhood information aggregation, which restricts their ability to capture global topological structures underlying tissue‐wide organization. To address these limitations, we propose STransformer, a versatile deep learning framework capable of processing both spatial multi‐modal and multi‐omics data. By synergistically integrating a GCN‐based module with a Transformer architecture, STransformer simultaneously captures short‐range local cellular interactions and long‐range global tissue organization, thereby achieving superior performance in resolving intricate tissue heterogeneity.

Through comprehensive evaluations, STransformer demonstrates exceptional capability in deciphering tissue heterogeneity across a diverse range of species and tissue types. When applied to spatial multi‐modal data, STransformer accurately identifies canonical spatial domains and effectively denoises gene expression profiles in the DLPFC. Moreover, comparative analysis of healthy and Alzheimer's disease slices reveals mechanistic insights into the transition from normal to disease states. For spatial multi‐omics applications, STransformer successfully dissects intricate immune microenvironments within the human tonsil, revealing a high degree of synergistic regulation between transcriptomic and proteomic expression. Furthermore, by jointly analyzing spatial epigenomic and transcriptomic data, it accurately infers region‐specific regulatory mechanisms in the mouse embryonic brain. Finally, in time‐series spatiotemporal data of the chicken embryonic heart, STransformer successfully captures the dynamic spatiotemporal trajectories of cardiogenesis, highlighting the progressive spatial differentiation of cardiac regions during development.

Despite rapid advancements in spatial technologies, current spatial data remain susceptible to technical noise and limited molecular capture. Looking ahead, continued innovations in spatial technologies are expected to substantially improve the resolution, coverage, and signal‐to‐noise ratio of spatial measurements. Meanwhile, as spatial technologies generate increasingly large‐scale datasets, the standard self‐attention mechanism in STransformer may lead to increased memory consumption and runtime due to its quadratic computational complexity with respect to the number of spatial spots. Future work will focus on improving scalability by incorporating efficient attention mechanisms and memory‐efficient training schemes. With higher‐fidelity datasets and improved computational efficiency, STransformer is expected to enable a more refined and efficient dissection of tissue heterogeneity, further advancing our understanding of complex biological mechanisms.

## 4 Experimental Section

### 4.1 Data Description

We collect publicly available spatial multi‐modal and multi‐omics datasets from multiple platforms to comprehensively evaluate STransformer (Table S3). The DLPFC dataset, generated via the 10× Visium platform, comprises 12 slices ranging from 3460 to 4789 spots per slice. The MTG dataset consists of a healthy control slice with 4701 spots and an Alzheimer's disease slice with 4832 spots. The time‐series chicken heart dataset spanning three developmental stages (Day 7, Day 10, and Day 14) contains 494, 1039, and 1967 spots, respectively. In addition, the human tonsil dataset, generated using 10× Visium platform with protein co‐profiling technology, contains 4518 spots. The MISAR‐seq platform provides an embryonic mouse brain dataset at stage E15.5, comprising 1949 spots. Manual annotations for all datasets are obtained from their respective original studies.

### 4.2 Data Preprocessing

Prior to model training, all spatial multi‐modal and multi‐omics data are subjected to a standardized preprocessing procedure.

For the spatial transcriptomics data, the spatial gene expression matrix is processed using SCANPY [36]. Genes detected in fewer than 50 spatial spots or with total counts below 10 are excluded. The filtered gene expression matrix is subsequently normalized by library size. Lastly, highly variable genes (n = 2000) are selected using the Seurat v3 method [37], followed by standardization.

For the histology image data, we adopt the image feature extraction approach inspired by STAIG [11], in which BYOL [38] with a ResNet50 [39] backbone is used for self‐supervised feature learning, enabling extraction of robust features without negative samples. Briefly, tissue histology images are divided into patches centered at the spatial coordinates of each spot, ensuring alignment between image patches and spatial transcriptomic measurements. Each patch is converted to a grayscale image through simple filtering and subsequently fed into the BYOL framework, producing a 2048‐dimensional feature vector for each spot.

For spatial epigenomic data, peaks are selected based on spatial variability quantified by Moran's I [40]. The top 2000 most spatially variable peaks are retained and subsequently standardized.

For spatial proteomic data, protein features are directly standardized without additional feature selection owing to their relatively low dimensionality.

Consequently, we obtain a feature matrix $X_{i}\in R^{N_{spot}\times d_{i}}$ for each modality i, where $N_{spot}$ denotes the number of spatial spots and $d_{i}$ denotes the feature dimension.

### 4.3 Spatial Graph Construction

For each modality, we construct an undirected spatial graph G=(V,E). Each node in V represents a spatial spot with features given by the corresponding row of the preprocessed matrix $X_{i}$, while edges in E are constructed via a k‐nearest neighbor (KNN) strategy based on Euclidean distances derived from spatial coordinates, representing spatial neighborhood relationships (Note S2). The adjacency matrix A is defined as:

(1) $$A_{ij}=\left\{\begin{array}{cc} 1, & j\in N_{k}\left(i\right)\;\text{or}\;i\in N_{k}\left(j\right),\\ 0, & \text{otherwise}, \end{array}\right.$$

where $N_{k}\left(i\right)$ denotes the set of k nearest neighbors of spot i. The resulting adjacency matrix is then normalized using the standard GCN renormalization scheme as follows:

(2) $$\hat{A}={\tilde{D}}^{-\frac{1}{2}}\tilde{A}{\tilde{D}}^{-\frac{1}{2}}$$

where $\tilde{A}=A+I$ is the adjacency matrix with self‐loops, and $\tilde{D}$ is the corresponding degree matrix. Furthermore, to improve model robustness and mitigate overfitting, we apply data augmentation during training by randomly dropping edges and masking node features.

### 4.4 Short‐Range Interaction Extraction

To capture short‐range spatial interactions among neighboring spots, we employ a residual spatial graph encoder based on GCN. By propagating information along the spatial graph topology, the encoder aggregates signals from local neighborhoods, enabling the extraction of localized cellular interactions within each modality. The encoder consists of a three‐layer GCN backbone, where node representations are iteratively updated through neighborhood aggregation. The layer‐wise propagation rule is defined as follows:

(3) $$H_{e}^{\left(l+1\right)}=\tanh\left(\hat{A}H_{e}^{\left(l\right)}W_{e}^{\left(l\right)}\right)$$

where $H_{e}^{\left(l\right)}$ denotes the node embedding at the l‐th layer (initialized as $H_{e}^{\left(0\right)}=X_{i}$), $W_{e}^{\left(l\right)}$ is the trainable weight matrix, and tanh(·) is the nonlinear activation function applied in hidden layers.

Formally, $F_{enc}\left(\cdot \right)$ denotes the composite transformation function parametrized by the stacked GCN layers, while $F_{res}\left(\cdot \right)$ denotes the residual mapping. The resulting modality‐aware representations $R_{i}\in R^{N_{spot}\times d_{latent}}$ are formulated as follows:

(4) $$R_{i}=F_{enc}\left(X_{i},\hat{A}\right)+F_{res}\left(X_{i},\hat{A}\right)$$

The residual mapping preserves modality‐specific input information during neighborhood aggregation, thereby maintaining spot‐level discriminative features and mitigating potential over‐smoothing caused by repeated graph propagation.

### 4.5 Long‐Range Tissue‐Wide Dependency Learning

In order to integrate modality‐specific representations and capture long‐range tissue‐wide dependencies, we employ a Transformer‐based architecture. Specifically, we construct a unified token sequence $S\in R^{\left(M\cdot N_{spot}\right)\times d_{latent}}$ by concatenating the modality‐aware representations from M distinct modalities. This process is mathematically formulated as follows:

(5) $$S=\overset{M}{\underset{i=1}{⨁}}R_{i}$$

where ⨁ denotes the concatenation operation and M represents the total number of input modalities. This unified sequence S is linearly projected to the initial embedding $E^{\left(0\right)}$ and then processed by the Transformer encoder, which utilizes Multi‐Head Self‐Attention (MSA) to model long‐range dependencies across all spots and modalities. For the l‐th encoder layer (1≤l≤N), the output $E^{\left(l\right)}$ is computed as follows:

(6) $${\tilde{E}}^{\left(l\right)}=\text{LayerNorm}\left(E^{\left(l-1\right)}+\text{MSA}\left(E^{\left(l-1\right)}\right)\right)$$

(7) $$E^{\left(l\right)}=\text{LayerNorm}\left({\tilde{E}}^{\left(l\right)}+\text{FFN}\left({\tilde{E}}^{\left(l\right)}\right)\right)$$

where FFN(·) and LayerNorm(·) denote Feed‐Forward Networks and Layer Normalization, respectively. Additionally, dropout is employed after each MSA and FFN sublayer for regularization.

Subsequently, the Transformer decoder is employed to further refine these representations, where $D^{\left(0\right)}$ is initialized from the encoder output $E^{\left(N\right)}$. Incorporating a Multi‐Head Cross‐Attention (MCA) mechanism to integrate contextual information, the update rule for the l‐th decoder layer (1≤l≤N) is formulated as follows:

(8) $${\hat{D}}^{\left(l\right)}=\text{LayerNorm}\left(D^{\left(l-1\right)}+\text{MSA}\left(D^{\left(l-1\right)}\right)\right)$$

(9) $${\tilde{D}}^{\left(l\right)}=\text{LayerNorm}\left({\hat{D}}^{\left(l\right)}+\text{MCA}\left({\hat{D}}^{\left(l\right)},E^{\left(N\right)}\right)\right)$$

(10) $$D^{\left(l\right)}=\text{LayerNorm}\left({\tilde{D}}^{\left(l\right)}+\text{FFN}\left({\tilde{D}}^{\left(l\right)}\right)\right)$$

where ${\hat{D}}^{\left(l\right)}$ acts as the query and the encoder output $E^{\left(N\right)}$ provides the keys and values. Similarly, dropout is applied to the outputs of each MSA, MCA, and FFN sublayer for regularization.

The decoder output $D^{\left(N\right)}$ is linearly projected and activated, resulting in a $d_{output}$‐dimensional representation sequence $H_{o}\in R^{\left(M\cdot N_{spot}\right)\times d_{output}}$, which is expressed as follows:

(11) $$H_{o}=\sigma \left(D^{\left(N\right)}W_{out}\right)$$

where $W_{out}$ is the learnable weight matrix, σ(·) denotes the sigmoid activation function, and $d_{output}$ denotes the dimension of the latent space.

To synthesize cross‐modal information, the representation sequence $H_{o}$ is split into modality‐specific components $\left\{Z_{1},Z_{2},\cdots ,Z_{M}\right\}$, where each $Z_{i}\in R^{N_{spot}\times d_{output}}$. The final latent spatial representation Z is defined as the arithmetic mean of these components:

(12) $$Z=\frac{1}{M}\sum_{i=1}^{M}Z_{i}$$

### 4.6 Feature Reconstruction

To preserve essential biological information within the latent space, we employ a three‐layer graph decoder to reconstruct the original molecular features from the $Z_{i}$ for each modality i. For the l‐th decoding layer, the layer‐wise propagation rule is defined as follows:

(13) $$H_{d}^{\left(l+1\right)}=\tanh\left(\hat{A}H_{d}^{\left(l\right)}W_{d}^{\left(l\right)}\right)$$

where $H_{d}^{\left(0\right)}=Z_{i}$, and $W_{d}^{\left(l\right)}$ denotes the trainable weight matrix.

The final output of the decoder generates the reconstructed feature matrix $\hat{X_{i}}$, which serves as the basis for the reconstruction loss function (detailed in Section 4.7) to optimize the model parameters.

### 4.7 Joint Objective Function Formulation

We employ a joint objective function that balances feature reconstruction across all modalities with geometric consistency between the original and reconstructed features. Specifically, we calculate the Mean Squared Error (MSE) between the input $X_{i}$ and the reconstructed features ${\hat{X}}_{i}$ across all M available modalities. For the i‐th modality, the reconstruction loss is formulated as follows:

(14) $$L_{rec}^{\left(i\right)}={∥X_{i}-{\hat{X}}_{i}∥}_{2}^{2}$$

where ${∥\cdot ∥}_{2}$ denotes the $L_{2}$ norm.

Additionally, we enforce directional consistency across all modalities using cosine similarity, ensuring geometric alignment between the original input $X_{i}$ and the reconstructed feature ${\hat{X}}_{i}$. For the i‐th modality, the consistency loss is defined as follows:

(15) $$L_{\cos}^{\left(i\right)}=1-\frac{\left\langle X_{i},{\hat{X}}_{i}\right\rangle }{∥X_{i}{∥}_{2}{∥{\hat{X}}_{i}∥}_{2}}$$

where ⟨·,·⟩ denotes the dot product between vectors.

Overall, the final total objective function is formulated as follows:

(16) $$L=\sum_{i=1}^{M}\lambda_{rec}^{\left(i\right)}L_{rec}^{\left(i\right)}+\sum_{i=1}^{M}\lambda_{\cos}^{\left(i\right)}L_{\cos}^{\left(i\right)}$$

Here, $\lambda_{rec}^{\left(i\right)}$ and $\lambda_{\cos}^{\left(i\right)}$ are hyperparameters that balance the contributions of each modality to the overall objective. A sensitivity analysis of these loss‐weight hyperparameters is provided in Figure S5.

### 4.8 Implementation and Baseline Comparison

STransformer is developed using the PyTorch framework within a Python environment. For reproducibility, the complete source code is publicly available. To demonstrate the efficacy of STransformer in spatial clustering, we benchmark it against the methods listed below, all of which are implemented using official implementations with recommended settings.

- *STAGATE* [6]. STAGATE adopts a graph attention auto‐encoder to learn low‐dimensional latent embeddings. By leveraging an attention mechanism to adaptively aggregate information from spatial neighbors, the method generates discriminative latent embeddings for spatial clustering. In this method, the default parameter settings are as follows: *n_epochs* = 500, *lr* = 0.001, *n_comps* = 15, and *pre_resolution* = 0.2.
- *stLearn* [9]. stLearn combines transcriptomic profiles with histological information. It employs a pre‐trained deep neural network to extract morphological features and smooths gene expression by incorporating morphological similarity alongside spatial distance. In this method, *n_comps* and *weights* are set to 15 and *“physical_distance*”, respectively.
- *GraphST* [8]. GraphST leverages graph self‐supervised contrastive learning to extract informative features. It employs a GNN encoder to capture local spatial dependencies and optimizes latent embeddings by contrasting positive spatial pairs against negative pairs, facilitating accurate spatial clustering and integration. The default parameters are used in our study: *epochs* = 600 and *k* = 5.
- *SEDR* [7]. SEDR employs a masked deep autoencoder to reconstruct gene expression and a variational graph autoencoder to capture spatial topology. The method jointly optimizes these networks to learn a compact latent representation that enhances spatial clustering performance. The training configuration adopts the default settings: *epochs* = 200, *lr* = 0.01, and *p_drop* = 0.2.
- *DeepST* [10]. DeepST integrates spatial location, gene expression, and morphological information. It employs a graph autoencoder coupled with a denoising autoencoder to extract latent representations, effectively minimizing data noise while preserving local spatial correlations for accurate domain identification. This experiment is conducted using the following default parameter settings: *pca_n_comps* = 200, *pre_epochs* = 800, *epochs* = 1000, *Conv_type* = *‘GCNConv’*.
- *STAIG* [11]. STAIG introduces an image‐aided graph contrastive learning framework. By leveraging deep morphological embeddings with a graph contrastive optimization strategy, STAIG effectively captures both transcriptomic and visual tissue heterogeneity for identifying spatial domains. In our experiment, we adopt the default settings: *epochs* = 300, *learning_rate* = 0.0005, *weight_decay* = 0.0001.
- *stDCL* [3]. stDCL employs a dual graph contrastive learning approach to process spatial transcriptomics data. It constructs a graph embedding autoencoder that fuses gene expression and spatial information, optimizing the representation learning to effectively capture distinct tissue regions. In our experiments, we use the default parameter settings: *prelr* = 0.001, *lr* = 0.005, *pre_epochs* = 500, and *epochs* = 1000.
- *COSMOS* [13]. COSMOS is designed for the cooperative integration of spatially resolved multi‐omics data. It extracts complementary features from different omics layers and integrates them into a unified embedding with modality weights determined by a weighted nearest neighbor strategy. The default parameters are used in our study: *wnn_epochs* = 500, *total_epochs* = 1000, and *lr* = 0.001.
- *SpatialGlue* [12]. SpatialGlue is designed for spatial multi‐omics integration with a dual‐attention mechanism. It first captures modality‐specific information within each omics layer and then integrates cross‐omics relationships to learn joint representations for spatial domain identification. The training configuration adopts the default settings: *epochs* = 200, *lr* = 0.0001.

## Conflicts of Interest

The authors declare no conflicts of interest.

## Supporting information


**Supporting File**: advs75969‐sup‐0001‐SuppMat.pdf.

## Data Availability

The open‐source Python implementation of STransformer is available at https://github.com/xingyili/STransformer. All datasets utilized in this study are publicly accessible and can be downloaded from Zenodo via the following link: https://zenodo.org/records/19345129.
