# Supplementary material for: Accurately Deciphering Tissue Heterogeneity From Spatial Multi‐Modal and Multi‐Omics With STransformer
Source: Adv Sci (Weinh). 2026 Jun 9:e75969. Online ahead of print. doi: 10.1002/advs.75969 (PMC13336508; doi:10.1002/advs.75969)
Supplement: Supplementary file 1 — Supporting File: advs75969‐sup‐0001‐SuppMat.pdf. [file ADVS-9999-e75969-s001.pdf]

**Supporting information to**

# Accurately Deciphering Tissue Heterogeneity from Spatial Multi-modal and Multi-omics with STransformer

Xingyi Li[1,2,3*], Jialuo Xu[1], Gaoyuan Du[1], Xiangting Jia[1], Dongmin Zhao[1], Chunyan Zhou[1], Kexin Xiao[1], Jia Gu[3*], Junnan Zhu[4*], and Xuequn Shang[1*]

[1]School of Computer Science, Northwestern Polytechnical University, Xi'an, Shaanxi, 710129, China

[2]Research & Development Institute of Northwestern Polytechnical University in Shenzhen, Shenzhen, Guangdong, 518063, China

[3]Faculty of Data Science, City University of Macau, Macau, 999078, China

[4]State Key Laboratory of Multimodal Artificial Intelligence Systems, Institute of Automation, Chinese Academy of Sciences, Beijing, 100190, China

[*]E-mail: xingyili@nwpu.edu.cn (Xingyi Li); jiagu@cityu.edu.mo (Jia Gu); junnan.zhu@nlpr.ia.ac.cn (Junnan Zhu); shang@nwpu.edu.cn (Xuequn Shang)

# Supplementary Note

## Supplementary Note S1. Evaluation metrics

The Adjusted Rand Index (ARI)[1] is employed as the evaluation metric to measure the consistency between clustering results and manual annotations, which can be calculated as:

$$ARI = \frac{\sum_{ij}\binom{n_{ij}}{2} - \left[\sum_i\binom{a_i}{2}\sum_j\binom{b_j}{2}\right]/\binom{n}{2}}{\frac{1}{2}\left[\sum_i\binom{a_i}{2} + \sum_j\binom{b_j}{2}\right] - \left[\sum_i\binom{a_i}{2}\sum_j\binom{b_j}{2}\right]/\binom{n}{2}}$$

**Applied datasets:** DLPFC dataset, Alzheimer’s disease dataset, human tonsil dataset, and chicken heart dataset.

## Supplementary Note S2. K-means clustering

K-means clustering divides samples into K groups by minimizing the sum of squared distances between samples and their corresponding cluster centroids. Using the implementation in scikit-learn, K-means (random seed=42) is applied to learned latent embeddings for the initial clustering. To enhance spatial consistency, a label refinement procedure adjusts cluster assignments according to local grid topology, considering 6 neighbors for hexagonal grids or 4 for square grids. A label is updated only when fewer than half of the neighboring spots support the current assignment and an alternative cluster forms a strict majority (>50%).

**Applied datasets:** DLPFC dataset, Alzheimer’s disease dataset, human tonsil dataset, embryonic mouse brain dataset, and chicken heart dataset.

## Supplementary Note S3. Differential expression analysis

Differential gene expression analysis is performed to identify differentially expressed genes (DEGs) across spatial regions. Genes are ranked using the Wilcoxon rank-sum test implemented in

Scanpy. Genes with adjusted p-value < 0.05 and |log fold change|>1 are considered significant and used for downstream enrichment analyses.

**Applied datasets:** DLPFC dataset, embryonic mouse brain dataset, and chicken heart dataset.

## Supplementary Note S4. GO enrichment analysis

In this study, GO enrichment analysis is performed on DEGs using hypergeometric testing based on the Biological Process (BP) category of the Gene Ontology. Terms with p-value < 0.05 are considered significantly enriched.

**Applied datasets:** DLPFC dataset, embryonic mouse brain dataset, and chicken heart dataset.

## Supplementary Note S5. CellChat

Cell–cell communication analysis is performed using CellChat[2], which infers intercellular signaling networks by analyzing ligand–receptor interactions from the CellChatDB. Communication probabilities are estimated based on the law of mass action and aggregated to construct cell–cell communication networks between cell groups. Ligand–receptor interaction pairs and selected signaling pathways are further visualized to characterize intercellular signaling patterns.

**Applied datasets:** Alzheimer's disease dataset.

## Supplementary Note S6. Identification of region-associated peaks

Region-associated peaks are identified using a rank-based statistical test implemented in Scanpy[3], where peaks are ranked using the Wilcoxon rank-sum test across regions. For the target region, peaks with adjusted p-value < 0.05 are considered significantly associated with the target region.

**Applied datasets:** embryonic mouse brain dataset.

## Supplementary Note S7. Motif enrichment analysis

Motif enrichment analysis is performed on the top 500 region-associated peaks. Peak sequences are extracted from the mm10 mouse genome assembly using bedtools[4] (v2.27.1) and saved in FASTA format. The resulting sequences are then used as input for MEME-ChIP[5] (https://meme-suite.org/meme/tools/meme-chip) to identify enriched sequence motifs. The identified motifs are compared against the HOCOMOCO[6] v11 mouse motif database for annotation, while all other parameters are kept at their default settings.

**Applied datasets:** embryonic mouse brain dataset.

## Supplementary Note S8. Temporal expression trend analysis

Temporal relative gene expression analysis is performed to characterize the expression patterns of key ventricular maturation-related genes across developmental stages. For each developmental stage, the average expression of each selected gene is calculated within the STransformer-identified ventricular regions. The relative expression level is then computed as the log2 fold change of the compact left ventricle and trabecular left ventricle relative to the right ventricle. Positive values indicate higher expression in the corresponding ventricular region than in the right ventricle, whereas negative values indicate lower expression.

**Applied datasets: chicken heart dataset.**

## Supplementary Note S9. Correlation analysis

Transcriptional correlation analysis is performed to evaluate the similarity of gene expression profiles among spatial regions identified by STransformer. Pairwise Pearson correlation coefficients are calculated based on the average gene expression profiles of each region using the implementation in Scanpy[3]. The resulting correlation matrix is visualized to illustrate transcriptional relationships among regions.

**Applied datasets:** chicken heart dataset.

# Supplementary Figures

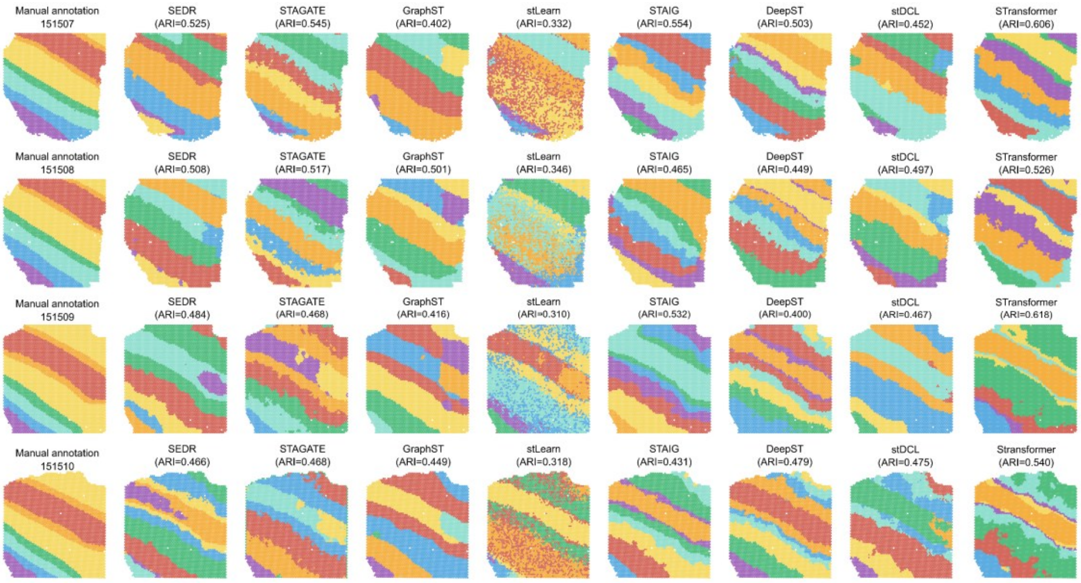


**Supplementary Figure S1. Evaluation on the DLPFC dataset.** Clustering performance of STransformer versus competing methods on DLPFC slices 151507–151510 in terms of ARI.

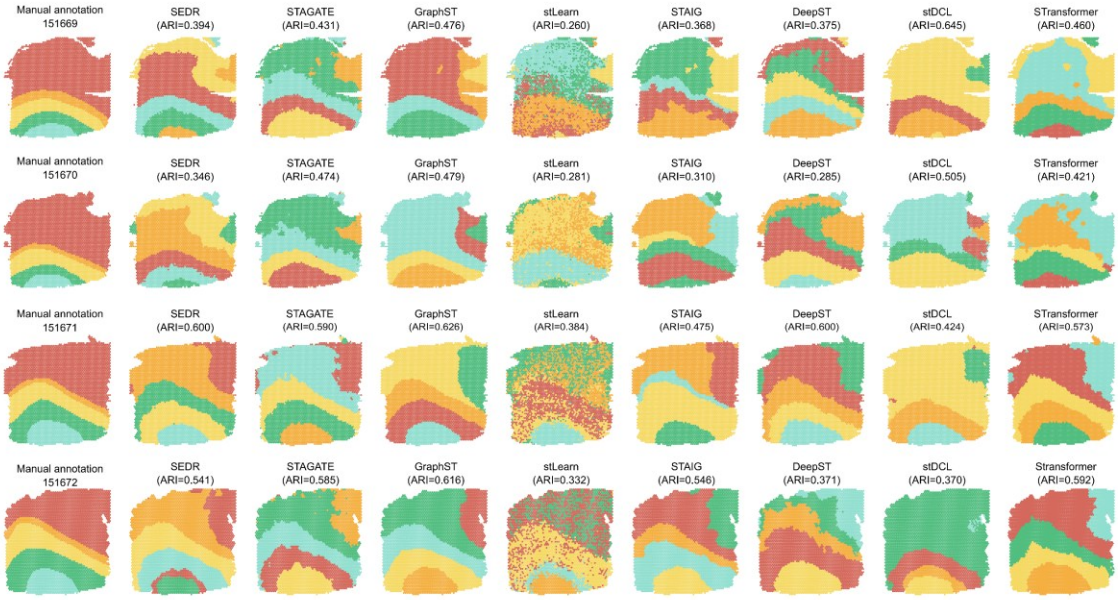


**Supplementary Figure S2. Evaluation on the DLPFC dataset.** Clustering performance of STransformer versus competing methods on DLPFC slices 151669–151672 in terms of ARI.

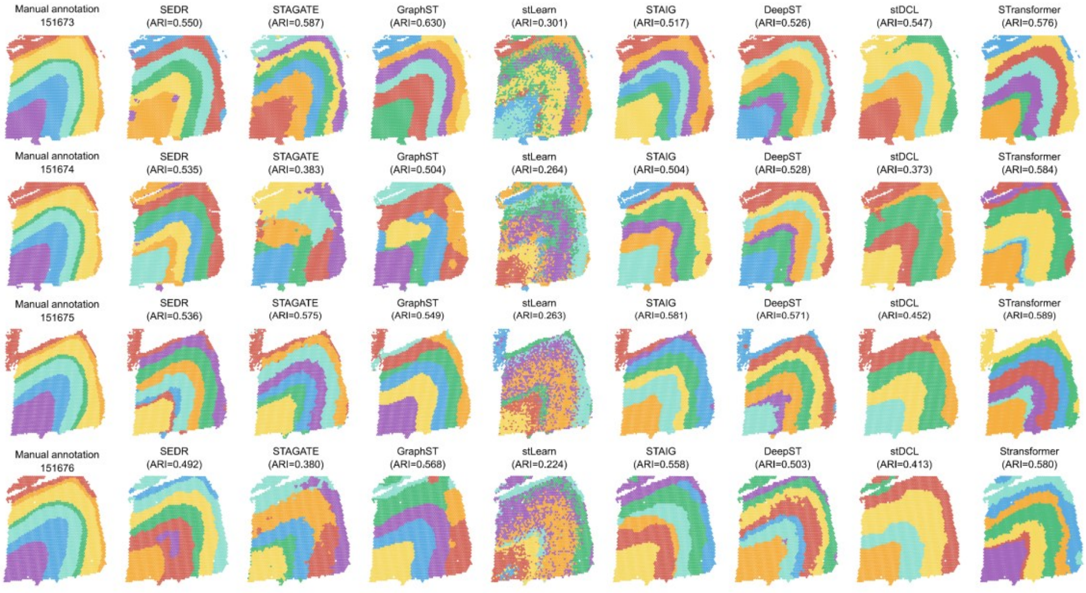


**Supplementary Figure S3. Evaluation on the DLPFC dataset.** Clustering performance of STransformer versus competing methods on DLPFC slices 151673–151676 in terms of ARI.

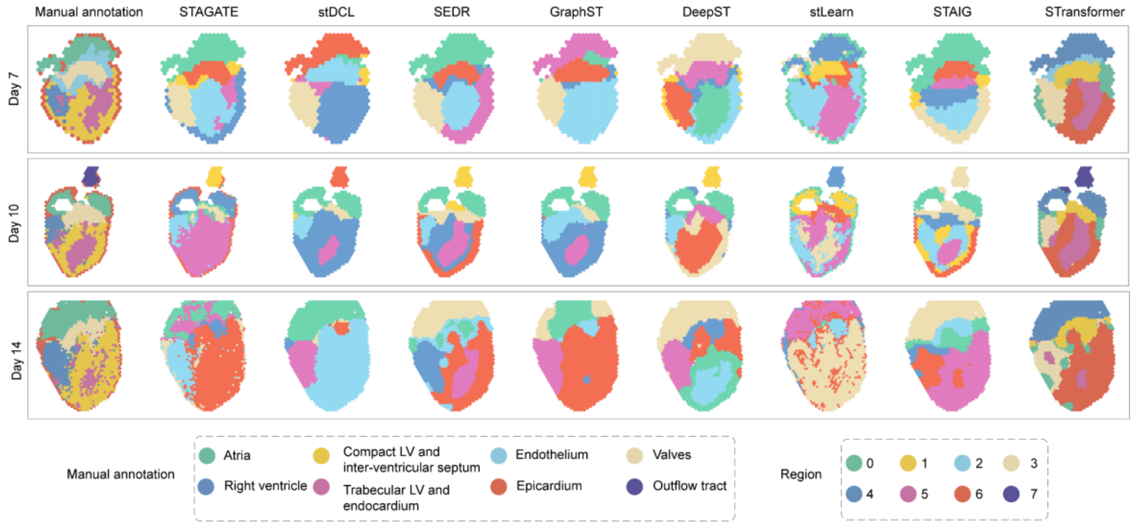


**Supplementary Figure S4. Evaluation on the chicken heart dataset.** This figure presents the clustering performance of seven competing methods on embryonic chicken heart slices at three distinct developmental stages (Day 7, Day 10 and Day 14), alongside the corresponding ground truth annotations.

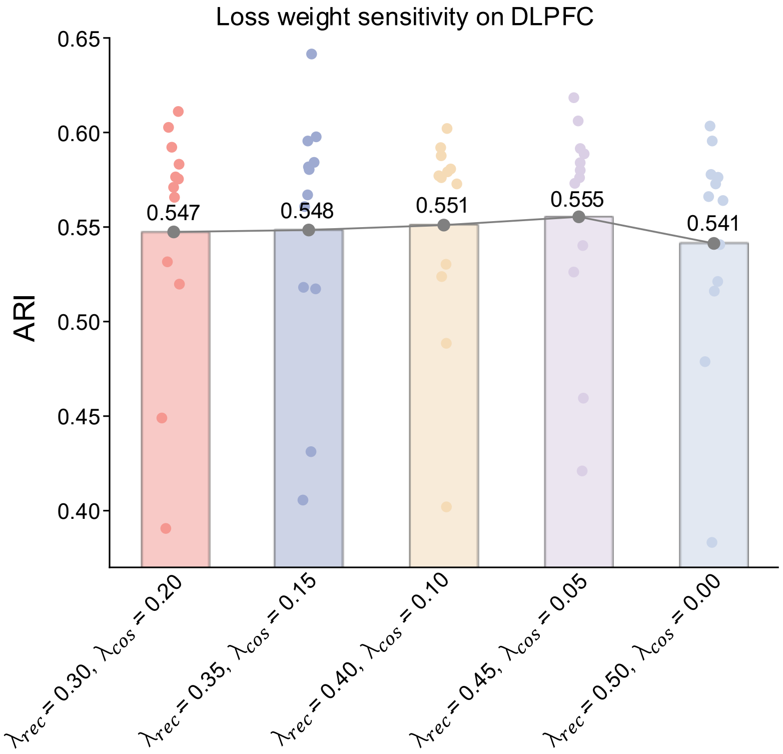


**Supplementary Figure S5. Sensitivity analysis of loss weights on the DLPFC dataset.** Bar plots show the mean ARI across 12 DLPFC slices under five different loss weight configurations. For each setting, $\lambda_{rec}^{(i)} = \lambda_{rec}$ and $\lambda_{cos}^{(i)} = \lambda_{cos}$ across modalities. Dots represent ARI values for individual slices, and the gray line connects the mean ARI values across different settings. The stable performance across these configurations indicates that STransformer is robust to variations in the hyperparameters used to balance reconstruction and consistency losses.

# Supplementary Tables

## Supplementary Table S1. Summary of representative spatial multi-modal and spatial multi-omics analysis methods.

| Method | Data type / applicability | Key characteristics | Strengths | Potential limitations / trade-offs |
|---|---|---|---|---|
| STAGATE[7] | Spatial transcriptomics | Graph attention autoencoder for spatial domain identification. | Capturing local spatial dependencies and improving spatial clustering. | Focusing mainly on spatial transcriptomics, with limited support for spatial multi-modal and multi-omics fusion. |
| SEDR[8] | Spatial transcriptomics | Masked autoencoder and variational graph autoencoder for spatial representation learning. | Combining a masked self-supervised autoencoder with a variational graph autoencoder to preserve transcriptional signals and spatial neighborhood information. | Focusing primarily on transcriptomic data; spatial multi-modal or multi-omics information is not explicitly modeled. |
| GraphST[9] | Spatial transcriptomics | Graph self-supervised learning framework for spatial representation learning. | Incorporating spatial neighborhood information through graph self-supervised learning to improve spatial embedding quality. | Relying mostly on local graph structure; long-range tissue-wide dependencies may be insufficiently captured. |
| stDCL[10] | Spatial transcriptomics | Dual graph contrastive learning for spatial representation learning. | Learning robust spatial embeddings through dual graph contrastive objectives for spatial domain identification. | Focusing on unimodal spatial transcriptomics and lacking explicit integration of spatial multi-modal and multi-omics data. |
| stLearn[11] | Spatial transcriptomics with histology | SME-based expression enhancement for integrating gene expression, spatial distance, and tissue morphology. | Using histological information to improve spatial analysis and biological interpretation. | Treating image-derived information mainly as auxiliary features rather than enabling deep cross-modal fusion. |
| DeepST[12] | Spatial transcriptomics with histology | Deep histology feature extraction and graph contrastive learning for spatial representation learning. | Enhancing spatial clustering by integrating histological context with gene expression and spatial information. | Being primarily designed for spatial transcriptomics and not specifically addressing spatial multi-omics integration. |
| STAIG[13] | Spatial transcriptomics with histology | Image-aided graph contrastive learning for spatial transcriptomics analysis. | Incorporating morphology-derived features to improve spatial domain identification and tissue structure characterization. | Focusing on transcriptome-image integration and showing limited applicability to general spatial multi-omics data. |
| SpatialGlue[14] | Spatial multi-omics | Graph neural network with dual-attention mechanisms for intra-omics and cross-omics integration. | Learning modality-specific importance through dual attention for intra-omics and cross-omics integration. | Relying mainly on graph-based local aggregation and potentially insufficiently capturing long-range tissue-wide dependencies. |
| COSMOS[15] | Spatial multi-omics | Modality-specific GCN encoding and WNN-derived modality weighting for spatial multi-omics integration. | Extracting complementary omics features and producing an integrated embedding for spatial domain identification. | Emphasizing local spatial dependencies while less explicitly modeling global tissue-wide organization. |
| SpaMV[16] | Spatial multi-omics | Graph attention network-based spatial multi-omics integration framework for interpretable multi-view representation learning. | Providing interpretable latent representations and supporting spatial domain clustering. | Depending on the consistency and complementarity of different omics views. |
| soFusion[17] | Spatial multi-omics | GCN-based intra-omics embedding and inter-omics feature learning for constructing joint spatial multi-omics representations. | Capturing both shared and modality-specific information to improve spatial domain identification. | Depending on the quality and compatibility of paired omics inputs. |

**Supplementary Table S2. Ablation Settings of STransformer in DLPFC.**

| Method | Graph encoder | Transformer | Graph decoder |
|---|---|---|---|
| STransformer | ✓ | ✓ | ✓ |
| w/o Graph encoder | ✗ | ✓ | ✓ |
| w/o Transformer | ✓ | ✗ | ✓ |
| w/o Graph decoder | ✓ | ✓ | ✗ |

**Supplementary Table S3. Description of all datasets used in this study.**

<table>
<tr><th>Platform</th><th>Tissue name</th><th>Slice</th><th>#Spots</th></tr>
<tr><td rowspan="18">10x Visium</td><td rowspan="12">Human dorsolateral prefrontal cortex (DLPFC)[18]</td><td>151507</td><td>4226</td></tr>
<tr><td>151508</td><td>4384</td></tr>
<tr><td>151509</td><td>4789</td></tr>
<tr><td>151510</td><td>4634</td></tr>
<tr><td>151669</td><td>3661</td></tr>
<tr><td>151670</td><td>3498</td></tr>
<tr><td>151671</td><td>4110</td></tr>
<tr><td>151672</td><td>4015</td></tr>
<tr><td>151673</td><td>3639</td></tr>
<tr><td>151674</td><td>3673</td></tr>
<tr><td>151675</td><td>3592</td></tr>
<tr><td>151676</td><td>3460</td></tr>
<tr><td rowspan="2">Human Middle Temporal Gyrus (MTG)[19]</td><td>Healthy control (CT)</td><td>4701</td></tr>
<tr><td>Alzheimer’s disease (AD)</td><td>4832</td></tr>
<tr><td>Human tonsil</td><td>S2</td><td>4518</td></tr>
<tr><td rowspan="3">Chicken heart[20]</td><td>Day 7</td><td>494</td></tr>
<tr><td>Day 10</td><td>1039</td></tr>
<tr><td>Day 14</td><td>1967</td></tr>
<tr><td>MISAR-seq</td><td>Embryonic mouse brain[21]</td><td>E15.5</td><td>1949</td></tr>
</table>

**Supplementary Table S4. Significant ligand-receptor (L-R) pairs detected in the healthy control (CT) group.**

| Index | Interaction name | Pathway name | Ligand | Receptor | Annotation |
|---|---|---|---|---|---|
| 1 | CCL2_ACKR1 | CCL | CCL2 | ACKR1 | Secreted Signaling |
| 2 | NPY_NPY1R | NPY | NPY | NPY1R | Secreted Signaling |
| 3 | NPY_NPY5R | NPY | NPY | NPY5R | Secreted Signaling |
| 4 | NPY_GPR83 | NPY | NPY | GPR83 | Secreted Signaling |
| 5 | PDYN_OPRL1 | OPIOID | PDYN | OPRL1 | Secreted Signaling |
| 6 | PDYN_OPRM1 | OPIOID | PDYN | OPRM1 | Secreted Signaling |
| 7 | PENK_OPRL1 | OPIOID | PENK | OPRL1 | Secreted Signaling |
| 8 | PENK_OPRM1 | OPIOID | PENK | OPRM1 | Secreted Signaling |
| 9 | SST_SSTR1 | SOMATOSTATIN | SST | SSTR1 | Secreted Signaling |
| 10 | SST_SSTR2 | SOMATOSTATIN | SST | SSTR2 | Secreted Signaling |
| 11 | CORT_SSTR1 | SOMATOSTATIN | CORT | SSTR1 | Secreted Signaling |
| 12 | CORT_SSTR2 | SOMATOSTATIN | CORT | SSTR2 | Secreted Signaling |
| 13 | TAC1_TACR1 | TAC | TAC1 | TACR1 | Secreted Signaling |
| 14 | CRH_CRHR1 | CRH | CRH | CRHR1 | Secreted Signaling |
| 15 | SLITRK2_PTPRS | SLITRK | SLITRK2 | PTPRS | Secreted Signaling |
| 16 | SLITRK4_PTPRS | SLITRK | SLITRK4 | PTPRS | Secreted Signaling |

**Supplementary Table S5. Significant L-R pairs detected in the Alzheimer's disease (AD) group.**

| Index | Interaction name | Pathway name | Ligand | Receptor | Annotation |
|---|---|---|---|---|---|
| 1 | NRG1_ERBB4 | NRG | NRG1 | ERBB4 | Secreted Signaling |
| 2 | NRG2_ERBB4 | NRG | NRG2 | ERBB4 | Secreted Signaling |
| 3 | FGF9_FGFR3 | FGF | FGF9 | FGFR3 | Secreted Signaling |
| 4 | VEGFA_VEGFR1 | VEGF | VEGFA | FLT1 | Secreted Signaling |
| 5 | CCL2_ACKR1 | CCL | CCL2 | ACKR1 | Secreted Signaling |
| 6 | CXCL9_ACKR1 | CXCL | CXCL9 | ACKR1 | Secreted Signaling |
| 7 | CXCL10_ACKR1 | CXCL | CXCL10 | ACKR1 | Secreted Signaling |
| 8 | CCL2_ACKR2 | CCL | CCL2 | ACKR2 | Secreted Signaling |
| 9 | EDN1_EDNRB | EDN | EDN1 | EDNRB | Secreted Signaling |
| 10 | NPY_NPY5R | NPY | NPY | NPY5R | Secreted Signaling |
| 11 | NPY_GPR83 | NPY | NPY | GPR83 | Secreted Signaling |
| 12 | PDYN_OPRD1 | OPIOID | PDYN | OPRD1 | Secreted Signaling |
| 13 | PDYN_OPRM1 | OPIOID | PDYN | OPRM1 | Secreted Signaling |
| 14 | PENK_OPRD1 | OPIOID | PENK | OPRD1 | Secreted Signaling |
| 15 | PENK_OPRM1 | OPIOID | PENK | OPRM1 | Secreted Signaling |
| 16 | SST_SSTR1 | SOMATOSTATIN | SST | SSTR1 | Secreted Signaling |
| 17 | CORT_SSTR1 | SOMATOSTATIN | CORT | SSTR1 | Secreted Signaling |
| 18 | TAC1_TACR1 | TAC | TAC1 | TACR1 | Secreted Signaling |
| 19 | PRSS3_PARD3 | PARs | PRSS3 | PARD3 | Secreted Signaling |
| 20 | VIP_VIPR2 | VIP | VIP | VIPR2 | Secreted Signaling |
| 21 | KITL_KIT | KIT | KITLG | KIT | Secreted Signaling |
| 22 | PTHLH_PTH1R | PTH | PTHLH | PTH1R | Secreted Signaling |
| 23 | SLITRK1_PTPRD | SLITRK | SLITRK1 | PTPRD | Secreted Signaling |
| 24 | SLITRK1_PTPRS | SLITRK | SLITRK1 | PTPRS | Secreted Signaling |
| 25 | SLITRK4_PTPRS | SLITRK | SLITRK4 | PTPRS | Secreted Signaling |
| 26 | PPIA_BSG | CypA | PPIA | BSG | Secreted Signaling |

# Supplementary References


[1] Steinley, Douglas. "Properties of the hubert-arable adjusted rand index." Psychological methods 9.3 (2004): 386.

[2] Jin, Suoqin, et al. "Inference and analysis of cell-cell communication using CellChat." *Nature communications* 12.1 (2021): 1088.

[3] Wolf, F. Alexander, Philipp Angerer, and Fabian J. Theis. "SCANPY: large-scale single-cell gene expression data analysis." *Genome biology* 19.1 (2018): 15.

[4] Quinlan, Aaron R., and Ira M. Hall. "BEDTools: a flexible suite of utilities for comparing genomic features." *Bioinformatics* 26.6 (2010): 841-842.

[5] Machanick, Philip, and Timothy L. Bailey. "MEME-ChIP: motif analysis of large DNA datasets." *Bioinformatics* 27.12 (2011): 1696-1697.

[6] Kulakovskiy, Ivan V., et al. "HOCOMOCO: expansion and enhancement of the collection of transcription factor binding sites models." *Nucleic acids research* 44.D1 (2016): D116-D125.

[7] Dong, Kangning, and Shihua Zhang. "Deciphering spatial domains from spatially resolved transcriptomics with an adaptive graph attention auto-encoder." *Nature communications* 13.1 (2022): 1739.

[8] Xu, Hang, et al. "Unsupervised spatially embedded deep representation of spatial transcriptomics." *Genome Medicine* 16.1 (2024): 12.

[9] Long, Yahui, et al. "Spatially informed clustering, integration, and deconvolution of spatial transcriptomics with GraphST." *Nature communications* 14.1 (2023): 1155.

[10] Yu, Zhuohan, et al. "Accurate spatial heterogeneity dissection and gene regulation interpretation for spatial transcriptomics using dual graph contrastive learning." *Advanced Science* 12.3 (2025): 2410081.

[11] Pham, Duy, et al. "Robust mapping of spatiotemporal trajectories and cell–cell interactions in healthy and diseased tissues." *Nature communications* 14.1 (2023): 7739.

[12] Xu, Chang, et al. "DeepST: identifying spatial domains in spatial transcriptomics by deep learning." *Nucleic acids research* 50.22 (2022): e131-e131.

[13] Yang, Yitao, et al. "STAIG: Spatial transcriptomics analysis via image-aided graph contrastive learning for domain exploration and alignment-free integration." *Nature Communications* 16.1

(2025): 1067.

[14] Long, Yahui, et al. "Deciphering spatial domains from spatial multi-omics with SpatialGlue." *Nature Methods* 21.9 (2024): 1658-1667.

[15] Zhou, Yuansheng, et al. "Cooperative integration of spatially resolved multi-omics data with COSMOS." *Nature communications* 16.1 (2025): 27.

[16] Liu, Yang, et al. "Interpretable spatial multi-omics data integration and dimension reduction with SpaMV." *Research Square* (2025): rs-3.

[17] Yu, Na, et al. "soFusion: facilitating tissue structure identification via spatial multi-omics data fusion." *Briefings in Bioinformatics* 26.5 (2025): bbaf513.

[18] Maynard, Kristen R., et al. "Transcriptome-scale spatial gene expression in the human dorsolateral prefrontal cortex." *Nature neuroscience* 24.3 (2021): 425-436.

[19] Chen, Shuo, et al. "Spatially resolved transcriptomics reveals genes associated with the vulnerability of middle temporal gyrus in Alzheimer's disease." *Acta neuropathologica communications* 10.1 (2022): 188.

[20] Mantri, Madhav, et al. "Spatiotemporal single-cell RNA sequencing of developing chicken hearts identifies interplay between cellular differentiation and morphogenesis." *Nature communications* 12.1 (2021): 1771.

[21] Jiang, Fuqing, et al. "Simultaneous profiling of spatial gene expression and chromatin accessibility during mouse brain development." *Nature Methods* 20.7 (2023): 1048-1057.
